# Ancient diversification of eukaryotic MCM DNA replication proteins

**DOI:** 10.1186/1471-2148-9-60

**Published:** 2009-03-17

**Authors:** Yuan Liu, Thomas A Richards, Stephen J Aves

**Affiliations:** 1 Centre for Eukaryotic Evolutionary Microbiology, School of Biosciences, University of Exeter, Geoffrey Pope Building, Stocker Road, Exeter, EX4 4QD, UK; 2 School of Biosciences, University of Exeter, Geoffrey Pope Building, Stocker Road, Exeter, EX4 4QD, UK

## Abstract

**Background:**

Yeast and animal cells require six mini-chromosome maintenance proteins (Mcm2-7) for pre-replication complex formation, DNA replication initiation and DNA synthesis. These six individual MCM proteins form distinct heterogeneous subunits within a hexamer which is believed to form the replicative helicase and which associates with the essential but non-homologous Mcm10 protein during DNA replication. In contrast Archaea generally only possess one MCM homologue which forms a homohexameric MCM helicase. In some eukaryotes Mcm8 and Mcm9 paralogues also appear to be involved in DNA replication although their exact roles are unclear.

**Results:**

We used comparative genomics and phylogenetics to reconstruct the diversification of the eukaryotic Mcm2-9 gene family, demonstrating that Mcm2-9 were formed by seven gene duplication events before the last common ancestor of the eukaryotes. Mcm2-7 protein paralogues were present in all eukaryote genomes studied suggesting that no gene loss or functional replacements have been tolerated during the evolutionary diversification of eukaryotes. Mcm8 and 9 are widely distributed in eukaryotes and group together on the MCM phylogenetic tree to the exclusion of all other MCM paralogues suggesting co-ancestry. Mcm8 and Mcm9 are absent in some taxa, including *Trichomonas *and *Giardia*, and appear to have been secondarily lost in some fungi and some animals. The presence and absence of Mcm8 and 9 is concordant in all taxa sampled with the exception of *Drosophila *species. Mcm10 is present in most eukaryotes sampled but shows no concordant pattern of presence or absence with Mcm8 or 9.

**Conclusion:**

A multifaceted and heterogeneous Mcm2-7 hexamer evolved during the early evolution of the eukaryote cell in parallel with numerous other acquisitions in cell complexity and prior to the diversification of extant eukaryotes. The conservation of all six paralogues throughout the eukaryotes suggests that each Mcm2-7 hexamer component has an exclusive functional role, either by a combination of unique lock and key interactions between MCM hexamer subunits and/or by a range of novel side interactions. Mcm8 and 9 evolved early in eukaryote cell evolution and their pattern of presence or absence suggests that they may have linked functions. Mcm8 is highly divergent in all *Drosophila *species and may not provide a good model for Mcm8 in other eukaryotes.

## Background

DNA replication in eukaryotes is catalysed by a complex of proteins termed the replisome. Formation of the replisome occurs at multiple origins of replication and is a stepwise process co-ordinated by the cell cycle control machinery (reviewed in [1]). Central to the replisome in all phases of its genesis and function are MCM (mini-chromosome maintenance) proteins.

The genes encoding MCM proteins were first identified in budding and fission yeasts through various cell cycle mutant screens, most notably Bik Tye's *mcm *screen for mutants of *Saccharomyces cerevisiae *defective in the ability to propagate centromeric plasmids containing a single origin of replication [2]. The phenotype of many *mcm *mutants is dependent on the exact identity of the origin of replication, suggesting a defect affecting the initiation of DNA replication [3]. In *S. cerevisiae *six MCM genes, *MCM2–MCM7 *[4], were originally identified and have shared sequence characteristics suggesting homology and a history of paralogous duplication [5].

The MCM proteins form numerous and complex interactions, central to which is the formation of a hexamer, the Mcm2-7 complex [5,6]. Mcm2-7 is loaded on to the origin recognition complex (ORC) at the origin of replication prior to DNA replication, during late M and G1 phases of the cell cycle, to form the pre-replication complex (pre-RC). This is activated at the G1-S transition of the cell cycle by the assembly of further protein components, including the non-homologous Mcm10 protein, in response to cell cycle kinase activities (reviewed in [7]). Mcm10 associates with the Mcm2-7 hexamer in the active replisome and helps to stabilise DNA polymerase α-primase (reviewed in [8]). Mcm2-7, together with other accessory factors, is believed to act as the replicative helicase, unwinding DNA at the two replication forks to provide single-stranded template on which replicative synthesis can take place [9,10].

Genes encoding MCM helicases are also found in Archaea (although not in Eubacteria currently sampled) but, in contrast to eukaryotes, the archaeal helicase generally comprises a homohexamer formed from multiple protein copies encoded by a single MCM gene [11]. Mcm10 has no recognised homologue in Archaea. Two more members of the Mcm2-7 family have recently been described in eukaryotes: Mcm8 and Mcm9. Mcm8 functions in aspects of DNA replication in vertebrates [12-15] but not in *Drosophila*, where it has a meiotic role [16,17]; it is absent from nematodes and yeast. Less is known about Mcm9 [18] which is the largest of the Mcm2-9 paralogues, having a unique long C-terminal region [19]. It was originally thought that Mcm9 is vertebrate-specific as it is absent from *Drosophila*, nematodes and yeasts [19], but it is now recognised that Mcm8 and Mcm9 have a more widespread eukaryotic distribution [17,20,21]. Mcm1 is a transcription factor which is not directly involved in DNA replication and will not be considered here [22,23].

Our knowledge of MCM proteins and DNA replication is derived from research principally based on yeast and vertebrate animals. These taxa are members of the opisthokonts, which according to current taxonomic consensus is one of six eukaryotic 'supergroups' [24] and therefore only represents a relative small proportion of the evolutionary history and genomic diversity of known eukaryotic life. For the other five eukaryotic supergroups, studies of the DNA replisome or MCM protein function and diversity are limited [25]. Here we use comparative genomics and phylogenetic analysis to investigate the distribution of MCM DNA replication proteins across the eukaryotes, to reconstruct the evolutionary history of the Mcm2-9 proteins, and to gain insights into MCM functional diversification, in distantly related eukaryotic taxa and at the base of the eukaryotic tree of life.

## Results and discussion

### Identification of MCMs

**Figure 1:**
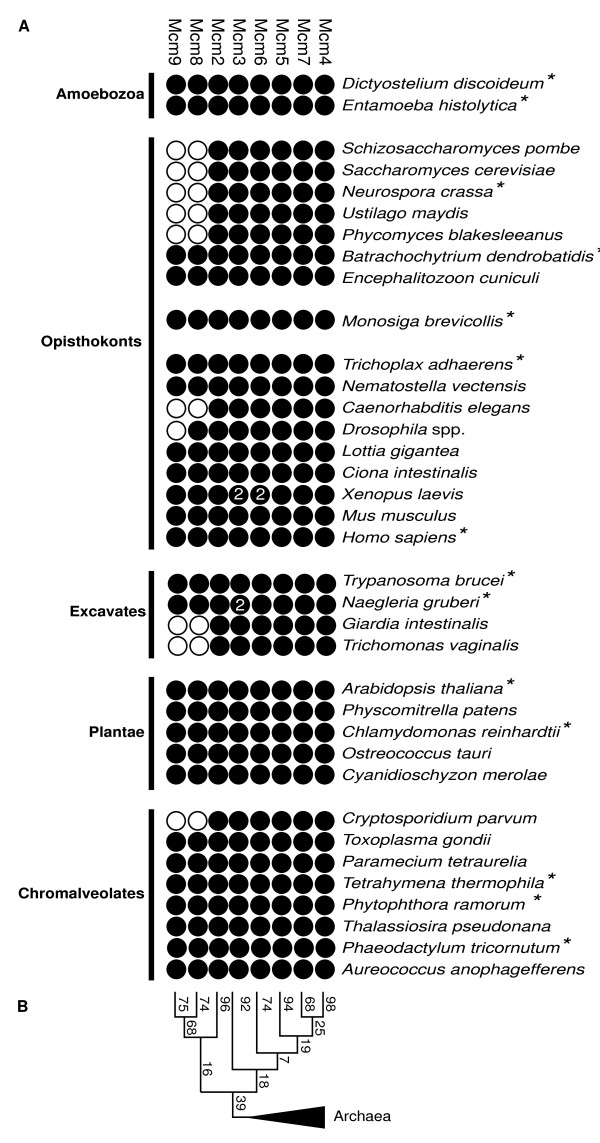
**Taxonomic distribution of Mcm2-9 and a summarised phylogenetic tree of MCMs**. This analysis shows that Mcm2-9 are present in the majority of the major eukaryotic 'supergroups' and that each MCM forms a moderate to strongly supported monophyletic group. Together these data demonstrate that Mcm2-9 were present in the last common eukaryotic ancestor. (A) Comparative genomic survey of MCM paralogues in 37 eukaryotic taxa. Mcm2-9 are shown on the x-axis and taxa are shown on the y-axis. Black circles indicate detections and open circles indicate no orthologues detected. Numbers within the black circles indicate the number of that specific MCM paralogue found in the taxa. Asterisks indicate species used for the "Noah's Ark" subset: for the results of the phylogenetic analysis see Additional file 5. (B) A summarised phylogenetic tree of MCMs based on Additional files 1, 2, 3, 4, emphasising the ML bootstrap support values for each MCM family, and relationships between each family which in most cases are weakly supported. The Archaea were used as an outgroup for reconstructing MCM phylogeny.

BLAST algorithms were used to identify MCM homologues encoded by the eukaryotic genomes of the 37 species listed in Table 1, covering five of the six eukaryotic supergroups [24,26-28] (Rhizaria unsampled). In *Naegleria *and *Xenopus *there were two proteins that grouped with Mcm3, and in *Xenopus *two Mcm6 proteins were found [29], providing the only examples of recent MCM duplication events among the taxa investigated. Comparative genomics and phylogenetic analysis (Fig. 1) showed that genes encoding six MCM proteins, Mcm2-7 homologues, were present in every eukaryotic genome sampled. These data suggest that the last common eukaryotic ancestor (LCEA) possessed all six Mcm2-7 paralogues and this ancient cell was therefore likely to contain a multi-subunit MCM protein complex composed of six paralogous proteins. These data suggest that an intricate and heterogeneous MCM protein complex evolved in an early phase of eukaryotic evolution.

**Table 1:** Taxonomic distribution of Mcm10 in eukaryotes.

**Supergroup**	**Rank**	**Species**	**Mcm10**	**Mcm8**	**Mcm9**
Amoebozoa	Dictyostelids	*Dictyostelium* *discoideum*	-	+	+

	Archamoebae	*Entamoeba* *histolytica*	-	+	+

Chromalveolates	Phaeophytes	*Aureococcus* *anophagefferens*	-	+	+

	Ciliates	*Paramecium* *tetraurelia*	-	+	+
		
		*Tetrahymena* *thermophila*	-	+	+

	Apicomplexa	*Cryptosporidium* *parvum*	-	-	-
		
		*Toxoplasma* *gondii*	55.m04882 (ToxoDB)	+	+

	Oomycetes	*Phytophthora* *ramorum*	Phyra1_1:82084 (JGI)	+	+

	Diatoms	*Phaeodactylum* *tricornutum*	Phatr2:32848 (JGI)	+	+
		
		*Thalassiosira* *pseudonana*	Thaps3:7942 (JGI)	+	+

Excavates	Heterolobosea	*Naegleria* *gruberi*	-	+	+

	Diplomonads	*Giardia* *lamblia*	-	-	-

	Parabasalids	*Trichomonas* *vaginalis*	XP_001314784	-	-

	Kinetoplastids	*Trypanosoma* *brucei*	XP_803462	+	+

Plantae	Land Plants	*Arabidopsis* *thaliana*	NP_179694	+	+
		
		*Physcomitrella* *patens*	Phypa1_1:183570 (JGI)	+	+

	Chlorophytes	*Chlamydomonas* *reinhardtii*	NP_001692606	+	+
		
		*Ostreococcus* *tauri*	Ostta4:34955 (JGI)	+	+

	Red Algae	*Cyanidioschyzon* *merolae*	-	+	+

Opisthokonts	Animals	*Caenorhabditis* *elegans*	NP_499456	-	-
		
		*Ciona* *intestinalis*	Cioin2:216242 (JGI)	+	+
		
		*Drosophila* *melanogaster*	NP_610097	+	-
		
		*Mus* *musculus*	NP_081566	+	+
		
		*Nematostella* *vectensis*	XP_001624863	-	-
		
		*Homo* *sapiens*	NP_877428*	+	+
		
			NP_060988*		
		
		*Lottia * *gigantea*	Lotgi1:238353 (JGI)	+	+
		
		*Trichoplax* *adhaerens*	Triad1:55391 (JGI)	+	+
		
		*Xenopus* *laevis*	NP_001082048	+	+

	Fungi	*Batrachochytrium* *dendrobatidis*	BDEG_01898 (BROAD)	+	+
		
		*Encephalitozoon* *cuniculi*	-	+	+
		
		*Neurospora* *crassa*	XP_960373	-	-
		
		*Saccharomyces* *cerevisiae*	NP_012116	-	-
		
		*Schizosaccharomyces* *pombe*	NP_596702	-	-
		
		*Phycomyces* *blakesleeanus*	Phybl1:72484 (JGI)	-	-
		
		*Ustilago* *maydis*	XP_758515	-	-

	Choanoflagellates	*Monosiga* *brevicollis*	Monbr1:32446 (JGI)	+	+

"+" indicates presence of an orthologue. "-" indicates that no orthologue was detected in the genome. "*" indicates that the genome encodes two isoforms of Mcm10.

Genes encoding the two most recently identified MCM family proteins, Mcm8 and Mcm9, were found to be present in the majority of eukaryotic genomes analysed and only absent in some opisthokonts, excavates and chromalveolates. Generally the Mcm8 and Mcm9 paralogues were either both present or both absent, suggesting that Mcm8 and Mcm9 may have associated functions. Of the taxa surveyed we found only one exception to this rule: *Drosophila *spp. possess Mcm8 but lack Mcm9.

BLAST searches and local Pfam searches of Mcm10 homologues showed that Mcm10 was also widely distributed among the eukaryotes sampled (Table 1). Mcm10 is conserved in opisthokonts and Plantae, except in *Encephalitozoon *and *Cyanidioschyzon *which represent eukaryotes with relatively small genomes and often encode divergent protein sequences [30,31]. Mcm10 proteins exhibit greater sequence divergence than Mcm2-9 orthologues and we cannot rule out that some Mcm10 orthologues may have fallen below detection limits employed here, however the distribution pattern of Mcm10 in representatives of different eukaryotic supergroups does not correlate with that of Mcm8 and Mcm9 (Table 1).

### The phylogeny and evolution of eukaryotic Mcm2-9

A phylogeny of the full Mcm2-9 protein family was reconstructed from the amino acid alignments of each conserved MCM domain [5,32] by performing a fast Maximum-likelihood (ML) analysis using PHYML [33] and a Bayesian analysis using MRBAYES [34]. Both phylogenetic analyses classified the MCM homologues as belonging to specific paralogue families and resolved the eukaryotic Mcm2-7 paralogues into six distinct monophyletic groups (Additional files 1, 2, 3, 4). Each MCM paralogous set was supported by moderate to high ML bootstrap values and Bayesian posterior probabilities (Mcm2: 96%/1.00 respectively; Mcm3: 92%/1.00; Mcm4: 98%/1.00; Mcm5: 94%/1.00; Mcm6: 74%/1.00; Mcm7: 68%/1.00) (Fig. 1B and Additional files 1, 2, 3). These results support the conclusion that all six Mcm2-7 proteins were present in the LCEA and that consecutive duplication, which formed these six defined MCM paralogues, occurred prior to the diversification of the sampled eukaryotic taxa.

Our phylogenetic analyses also grouped Mcm8 and Mcm9 into two distinct monophyletic groups supported by ML bootstrap values and Bayesian posterior probabilities (Mcm8: 74%/1.00; Mcm9: 75%/0.99) and placed Mcm8 and Mcm9 as sister paralogues to the exclusion of all other MCM paralogues (68% ML bootstrap support) (Fig. 1B and Additional file 4). This shared derived ancestry and linked pattern of presence and absence suggest that Mcm8 and Mcm9 have both co-function and distinct co-ancestry.

Both phylogenetic analyses failed to resolve the branching relationship between the MCM paralogues. To attempt to resolve these relationships a "Noah's Ark" dataset was analysed comprising a more limited sampling of eukaryotes from each major taxon (species indicated by asterisks in Fig 1); this also failed to yield resolution in the backbone of the tree (Additional file 5). The lack of resolution among these ancient paralogue groups may be the consequence of lack of signal within the relatively short alignment used for phylogenetic analyses (240 amino acid characters). This feature could also be a product of a hard polytomy or the rapid consecutive duplication of MCM parental forms into eight paralogues at the base of the eukaryotic tree resulting in limited availability of evolutionary signal to support the relative branching order of the MCM paralogues.

Previous studies on DNA replication of Archaea have already shown that the core replication machineries of the eukaryotes and Archaea possess fundamental similarities and have many homologous protein components [35], indicative of common ancestry. There is currently no known eubacterial homologue to the eukaryotic MCMs but we identified a single highly conserved MCM homologue in all the Archaea studied (except *Methanococcus jannaschii *which possesses four MCM proteins). Our phylogenetic analyses demonstrated that the four paralogues in the complete genome sequence of the euryarchaeote *M. jannaschii *can be best explained by a series of Archaea-specific gene duplications (98% ML bootstrap support) (Additional file 4) which occurred separately from the eukaryotic MCM gene duplications. Our analyses therefore suggest that the eukaryotic Mcm2-9 may be derived by gene duplication events from a single, archaeal-like, ancestral MCM and the diversification of this archaeal-like MCM would have given rise to Mcm2-9 encoded in the genome of the LCEA.

### The phylogeny and evolution of *Drosophila *Mcm8

**Figure 2:**
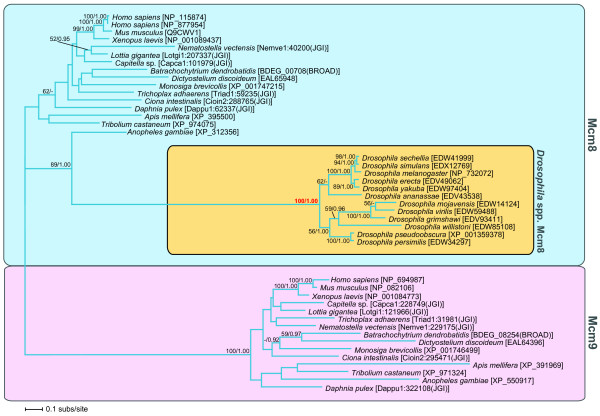
**Phylogenetic analysis of animal Mcm8 and Mcm9**. This analysis was conducted to investigate the diversification and branching position of the *Drosophila *Mcm8 proteins as *Drosophila *is the only lineage with an Mcm8 but with no Mcm9. The analysis shows that the *Drosophila *Mcm8 proteins are highly divergent. The phylogeny shown is a calculated from an amino acid alignment of 43 sequences and 366 characters. The tree topology shown was calculated using PHYML methods. Mcm8 paralogues are shown in blue and Mcm9 paralogues in pink. The orange coloured block indicates the relationship of *Drosophila *spp. Mcm8 with Mcm8 sequences from other animals plus additional outgroups. Numbers on nodes indicate the ML bootstraps and Bayesian posterior probabilities (bootstrap values below 50% and posterior probabilities below 0.90 are not shown). The phylogeny is shown as rooted on the Mcm9 paralogue.

*Drosophila melanogaster *was the only species sampled that did not show co-possession or co-absence of Mcm8 and Mcm9. Our general MCM phylogeny suggested that the *D. melanogaster *Mcm8 protein was highly divergent (Additional file 4). To further investigate the apparent loss of Mcm9 and radical divergence of Mcm8 in *Drosophila *species we conducted an animal Mcm8 and 9 phylogenetic analysis which included a sampling of all 12 *Drosophila *genomes and additional Insecta genome sequences, plus additional outgroups. The Mcm8 and Mcm9 comparative genomic analyses and phylogeny demonstrated the monophyletic groupings of Mcm8 and Mcm9 and confirmed the absence of Mcm9 in all the *Drosophila *species (100% ML bootstrap support and 1.00 Bayesian posterior probability; Fig. 2). This analysis also demonstrated that the *Drosophila *Mcm8 cluster formed an extremely long branch within the animal Mcm8 clade (100% ML bootstrap support and 1.00 Bayesian posterior probability; Fig. 2) suggesting a pattern of radical evolutionary change specifically in the *Drosophila *Mcm8 gene family. The divergence of *Drosophila *Mcm8 from other Mcm8 sequences may be related to the absence of Mcm9 in *Drosophila *or a radical change in functional role.

### Gain and loss of Mcm8 and Mcm9 in eukaryotic evolution

The comparative genomics studies of MCMs have revealed that Mcm8 and Mcm9 are widely distributed in (at least) five of the six eukaryotic supergroups and only absent in some opisthokonts, excavates and chromalveolates. By adapting current understandings on the phylogenetic and taxonomic groupings of the eukaryotes [26-28,36] it is possible to make some deductions about gain and loss events of Mcm8 and Mcm9 across the eukaryotic evolutionary tree. The presence of Mcm8 and Mcm9 in five of the six supergroups suggests that Mcm8 and Mcm9 also arose early in eukaryotic evolution. Mcm8 and Mcm9 were both absent from all analysed fungal genomes, with the exception of *Encephalitozoon *and *Batrachochytrium*. In comparison to the current consensus fungal phylogeny [37,38], these data suggest that the loss of Mcm8 and 9 in the fungi was probably a shared loss event in the last common ancestor of the ascomycetes, basidiomycetes and zygomycetes sampled. Further secondary loss events were identified in the Apicomplexa *Cryptosporidium *and in the animal *Caenorhabditis *lineages.

Mcm8 and 9 were absent in the excavates *Giardia *and *Trichomonas *which according to some schemes for the eukaryotic phylogeny are potentially the primary branch in the eukaryotic phylogeny [39]. Since the root of the eukaryotic tree is still unclear, when the Mcm8 and Mcm9 paralogues originated within the eukaryotic phylogeny remains a puzzle. However, a homologue of Mcm8 was detected as a partial sequence in Genome Survey Sequences (GSS) data for the diplomonad *Spironucleus barkhanus*, which is a close relative of *Giardia*. This suggests that Mcm8 and possibly Mcm9 originated before the common ancestor of *Giardia *and *Trichomonas *branched from the base of the eukaryotic tree and that Mcm8 and Mcm9 were also present in the LCEA. This identifies at least two additional cases of Mcm8-9 co-loss within the 'metamonads' [40,41] including one loss in the diplomonads (*Giardia*) and the parabasalids (*Trichomonas*). Together this suggests at least five separate cases of paired Mcm8 and 9 loss during the eukaryotic radiation, providing indirect support for the hypothesis that Mcm8 and 9 may have some currently unidentified interdependent functional roles.

### Reciprocity between MCM evolution and function studies

In most Archaea, a single MCM forms a homohexamer. A number of archaeal MCMs have been demonstrated to act as a helicase to unwind double-stranded DNA [35]. Helicase activity of eukaryotic MCMs has been found *in vitro *in a sub-complex of Mcm4, Mcm6 and Mcm7 [42] or in a super-complex of Mcm2-7 with Cdc45 and GINS accessory proteins [9,10]. The comparative genomics and phylogenetic analysis of MCMs have revealed that the LCEA possessed all six Mcm2-7 proteins, which seem to have diversified from a single archaeal-like, ancestral MCM. This suggests that the ancestral enzymatic activity of eukaryotic Mcm2-7 proteins is unwinding DNA as a processive helicase. Because both eukaryotes and Archaea have a functional complex of six individual MCMs it is likely that early eukaryotes also had a six-protein MCM functional complex. However, eukaryotes replaced a single gene six-protein complex with a six gene six-protein complex.

Mcm8 and Mcm9 have received relatively few functional studies. Mcm8 has been characterised recently in humans, *Xenopus *and *Drosophila *[12-17,43]. Human and *Xenopus *Mcm8 function in DNA replication, although there is no consensus regarding their exact role(s); early evidence suggested this may be in the elongation phase of DNA replication [12,13], but human Mcm8 has also been shown to associate and co-localise with Cdc6, an Mcm2-7 loading factor, implying a role in pre-RC formation [14,15]. The early evolutionary acquisitions of Mcm8 and Mcm9 suggest that Mcm8 in other eukaryotes might also have similar roles. The putative co-function of Mcm8 and Mcm9 as demonstrated by the pattern of co-presence, co-loss and distinct co-ancestry suggests that Mcm9 is also involved in DNA replication; this is supported by the recent demonstration that that *Xenopus *Mcm9 acts as a positive regulator of the Mcm2-7 loading factor Cdt1 and is required for assembly of pre-replication complexes [44]. Future comparative studies on Mcm8 and 9 functions will be informative.

In addition, a role in meiotic recombination has been identified for the *Drosophila *Mcm8, REC [16,17]. *Drosophila *was the only eukaryote analysed which has Mcm8 but lacks Mcm9 suggesting, if the co-function and distinct co-ancestry hypothesis is correct, that REC may have assumed the function of both proteins or acquired a novel function that does not require Mcm9 specifically in *Drosophila*. Mcm8 and Mcm9 are therefore likely to co-function in meiotic recombination in other eukaryotic lineages, but further work is required to investigate this hypothesis. The loss of Mcm8 and Mcm9 in some opisthokont lineages and *Cryptosporidium *could mean that Mcm8 and Mcm9 are dispensable for meiotic recombination or other unknown functions. Comparative genomics analyses of meiotic genes indicate that *Giardia *possesses several protein components required for meiosis [45,46]. Further studies should attempt to investigate and compare meiosis and Mcm8 and Mcm9 function between *Giardia *and other eukaryotes capable of sexual reproduction and which encode Mcm8 and Mcm9.

Functional studies on Mcm10 proteins suggest that Mcm10 is required for both DNA initiation and elongation phase of DNA replication [8]. In yeast and *Xenopus*, Mcm10 binds to both chromatin and the Mcm2-7 complex and recruits Cdc45 and DNA polymerase α-primase to replication origins [47-50]. Mcm10 forms part of the replisome complex that migrates away from replication origins during S-phase [50,51] and is required for stability and activity of the initiating polymerase, DNA polymerase α-primase [50,52]. Mcm10 homologues were found neither in Archaea nor in Eubacteria; the appearance of this protein seems to be unique within eukaryotes. The evolutionary pattern of Mcm10 (Table 1) indicates the importance and functional similarities of Mcm10 homologues within the eukaryotic genomes analysed.

## Conclusion

We have carried out a broad and deep phylogenetic analysis of MCM proteins involved in DNA replication. Eukaryote genomes encode up to nine such proteins all of which have an ancient origin and diversification within the eukaryotic lineage. Subunits of the Mcm2-7 hexamer appear to be universally present in eukaryotic taxa, as predicted by their likely DNA helicase role in DNA replication. Mcm2-9 distribution demonstrates diversification by gene duplication prior to the LCEA of extant eukaryotic supergroups. Mcm8 and 9 paralogues exhibit a distribution pattern characteristic of interdependent loss events in some taxa. Co-loss suggests related functions for Mcm8 and 9, which may play more peripheral roles in the cell than Mcm2-7. Finally, the non-homologous Mcm10 protein appears to be exclusively eukaryotic and exhibits greater sequence divergence across taxa than the Mcm2-9 family.

## Methods

### Sequence Data

We compared the DNA scaffold predicted transcriptome, and the annotated protein databases of 37 complete or draft eukaryotic genome sequence databases listed in Additional file 6.

In addition to the predicted eukaryotic protein databases, predicted protein databases of Archaea *Sulfolobus acidocaldarius*, *Thermofilum pendens*, *Methanococcus jannaschii*, *Archaeoglobus fulgidus*, *Thermoplasma volcanium*, *Pyrococcus horikoshii*, *Nanoarchaeum equitans *and *Halobacterium *sp. were also sampled to use as outgroups for the phylogenetic analyses. Archaeal genomes were available from the National Center for Biotechnology Information (NCBI) . To further confirm that no MCM homologues were detected in eubacterial genomes we also performed 'eubacterial'-restricted tBLASTn, BLASTp and PSI-BLAST searches of GenBank non-redundant sequence databases.

### Identifying Mcm2-7, Mcm8 and Mcm9 homologues

Homologous sequences of Mcm2 to Mcm7 proteins were identified by performing an all-against-all search of eukaryotic (listed in Additional file 6) and archaeal protein databases using the BLASTp algorithm with a cutoff expectation (E) value of 1 × 10^-20^. Candidates were tested by reciprocal BLAST searches. Mcm8 and Mcm9 homologues were checked by using both BLASTp and tBLASTn searches in BLAST options of the respective genome projects (listed in Additional file 6), with putative Mcm8 and Mcm9 protein sequences of closely related taxa as a BLAST seed for these sub-analyses. These multiple BLAST searches using paralogous genes as search seeds were conducted to ensure that all available paralogues were sampled from each of the target genomes. In cases of apparent absence, EST and GSS data of closely-related species were also searched. All candidate MCM proteins were checked by performing an NCBI Conserved Domain Database (CDD) search to confirm that all amino acid sequences sampled possessed an MCM domain. This analytical process recovered a number of protein sequences that were annotated as putative Mcm2-7 but were potentially misclassified paralogues. We therefore based our paralogue designation on phylogenetic analyses reported below.

### Identifying Mcm10 homologues

A search for Mcm10 homologues was performed with BLASTp and tBLASTn. The E-value of 1 × 10^-4 ^was used as cutoff to define Mcm10 homologous proteins. PSI-BLAST searches and local Pfam searches of eukaryotic databases were performed in order to identify more divergent Mcm10 homologues. PSI-BLAST searches were terminated after three iterations and revealed putative *Naegleria *Mcm10 (E-value = 3 × 10^-9 ^in PSI-BLAST iteration 2), *Trichomonas *Mcm10 (E-value = 1 × 10^-6 ^in PSI-BLAST iteration 2) and *Chlamydomonas *Mcm10 (E-value = 1 × 10^-37 ^in PSI-BLAST iteration 3), expanding the taxonomic distribution and evolutionary history of the Mcm10 protein family. Local Pfam searches were performed using the Hidden Markov Model (HMM) profile of Mcm10 domain available from Pfam (PF09332) [53] using the program HMMER ; no further potential Mcm10 homologues were detected.

### Eukaryotic Mcm2-9 phylogeny

The 290 putative MCM protein sequences were extracted and aligned using the multiple sequence alignment program MUSCLE [54] using the default settings. Putative *Ostreococcus *Mcm7 and *Aureococcus *Mcm8 had partial or comparatively 'noisy' sequences so were excluded from the analysis. The hypothetical *Plasmodium berghei *MCM genes were excluded because they were highly divergent and the sequences could not be aligned with confidence for the phylogenetic analysis. This process produced a multiple amino acid sequence alignment of 280 sequences which was manually corrected and then masked using the alignment editor SEAVIEW [55]. We masked the alignment prior to phylogenetic analyses to remove noisy or gappy regions and to focus the character sampling on the conserved MCM box region [5,32]. *Xenopus *zygotic Mcm3 [GenBank:Q7ZXZ0] and zygotic Mcm6 [GenBank:NP_001080590] were removed as they differed by only few amino acid residues from maternal Mcm3 and Mcm6 respectively after masking. This process produced an alignment of 278 sequences and 240 amino acid characters for phylogenetic analysis. Because of the size of this dataset we found that we could not execute the MODELGENERATOR [56] program even using relatively powerful computer platforms. Fast ML analyses were performed using the software PHYML with the WAG substitution matrix and the proportion of invariable sites (I) and eight category gamma distribution (Γ) estimated by PHYML [33]. 100 bootstrap replicates were calculated using PHYML to test topological support. To test alternative substitution matrices we repeated the analysis using the recently-developed LG matrix [57] which has been reported as an improvement on the WAG matrix. This analysis gave a similar result with slightly reduced bootstrap support values for the monophyly of the MCM paralogues (Additional file 7). Bayesian analysis was performed using MRBAYES [34], using the WAG substitution matrix as for the fast ML analysis. MRBAYES was run with two sets of four simultaneous Markov chains with a default temperature string and for 2 million generations and trees were sampled every 100 generations. The log likelihood output was compared across both runs and a burn-in of 5000 generation samples was excluded and a consensus phylogeny calculated from the remaining samples. The outputs of all analyses were viewed by the software TREEVIEW [58]. A "Noah's Ark" dataset was also analysed which comprised 14 eukaryotes (indicated by asterisks in Fig 1) sampled from each major taxon and included 120 sequences and an alignment sampling of 307 amino acid characters. MODELGENERATOR identified RtREV+Γ as the most appropriate substitution matrix with eight discrete categories and estimated the gamma distribution (Γ) parameter α as 0.49. 100 bootstrap replicates were calculated using PHYML.

### Animal Mcm8 and Mcm9 phylogeny

The general eukaryotic analyses demonstrate a potentially complicated pattern of gene loss for Mcm8 and Mcm9 across the animals. Homologues of *Capitella *sp., *Ciona intestinalis*, *Daphnia pulex*, *Tribolium castaneum*, *Anopheles gambiae*, *Apis mellifera*, *Mus musculus*, *Homo sapiens*, *Xenopus laevis*, *Lottia gigantea*, *Nematostella vectensis*, *Trichoplax adhaerens *(representing a wide taxonomic span of animals) and 12 *Drosophila *species were sampled and used for an additional animal-specific Mcm8 and Mcm9 phylogenetic analysis including 43 sequences and an alignment sampling of 366 amino acid characters. Amoebozoa *Dictyostelium*, the fungus *Batrachochytrium *and the choanoflagellate *Monosiga *Mcm8 and Mcm9 were included as outgroup taxa. This animal-specific Mcm8 and Mcm9 phylogeny was analysed using the same procedure as before. MODELGENERATOR identified RtREV+Γ as the most appropriate model with eight discrete categories and estimated the Γ parameter α as 0.93. Bootstrap analysis was carried out with 500 replicates. For the Bayesian analysis, we also analysed the Mcm8 and 9 phylogeny using MRBAYES with an RtREV+Γ model of sequence substitution. The Bayesian analysis was run as above but for one million generations with the first 400 generation samples discarded as burn-in. Both results were displayed using TREEVIEW.

## Authors' contributions

SJA and TAR conceived and planned the project. TAR designed the phylogenetic analysis. All authors contributed to design of bioinformatic analyses which were carried out by YL with supervision from TAR and SJA. All authors discussed the results, drafted and approved the final manuscript.

## Supplementary Material

Additional file 1 **Phylogenetic analysis of eukaryotic Mcm2-9 (part 1: Mcm4, Mcm7).** The tree was generated by fast ML analysis using PHYML and rooted with archaeal MCMs. The numbers on each node are the bootstrap values and posterior probabilities from Bayesian analysis (values below 50% and 0.90 are not shown). Supporting values for each MCM paralogue and for the relationships between the eight MCM paralogues are highlighted in red. Click here for file

Additional file 2 **Phylogenetic analysis of eukaryotic Mcm2-9 (part 2: Mcm5, Mcm6).** The tree was generated by fast ML analysis using PHYML and rooted with archaeal MCMs. The numbers on each node are the bootstrap values and posterior probabilities from Bayesian analysis (values below 50% and 0.90 are not shown). Supporting values for each MCM paralogue and for the relationships between the eight MCM paralogues are highlighted in red. Click here for file

Additional file 3 **Phylogenetic analysis of eukaryotic Mcm2-9 (part 3: Mcm3, Mcm2).** The tree was generated by fast ML analysis using PHYML and rooted with archaeal MCMs. The numbers on each node are the bootstrap values and posterior probabilities from Bayesian analysis (values below 50% and 0.90 are not shown). Supporting values for each MCM paralogue and for the relationships between the eight MCM paralogues are highlighted in red. Highlighted in green is the supporting value for the origin of eukaryotic MCMs. Click here for file

Additional file 4 **Phylogenetic analysis of eukaryotic Mcm2-9 (part 4: Mcm8, Mcm9, archaeal MCMs).** The tree was generated by fast ML analysis using PHYML and rooted with archaeal MCMs. The numbers on each node are the bootstrap values and posterior probabilities from Bayesian analysis (values below 50% and 0.90 are not shown). Supporting values for each MCM paralogue and for the relationships between the eight MCM paralogues are highlighted in red. Highlighted in green is the supporting value for the origin of eukaryotic MCMs. Click here for file

Additional file 5 **Treefile for "Noah's Ark" dataset phylogenetic analysis of eukaryotic Mcm2-9.** To attempt to further resolve the phylogeny of the Mcm2-9 proteins, specifically the branching relationships among the eight MCM paralogues, we conducted a reduced taxon phylogeny. In this analysis we used a limited sampling of eukaryotes from each major taxon (species indicated by asterisks in Figure 1) with the hope that the resulting reduction in tree space would enable us to resolve an improved phylogeny. The analysis did not show improved resolution among the terminal branches. The tree was calculated from an alignment of 120 sequences and 307 characters by fast ML analysis using PHYML with 100 bootstrap replicates. The tree can be viewed using TREEVIEW. Click here for file

Additional file 6 **Eukaryotic genomes and predicted proteomes analysed in this study.** Click here for file

Additional file 7 **Treefile for phylogenetic analysis of eukaryotic Mcm2-9 using LG matrix.** The tree was generated by fast ML analysis using PHYML with the LG matrix, with 100 bootstrap replicates. The tree can be viewed using TREEVIEW. Click here for file
