# Supplementary figures and images for: Ancient diversification of eukaryotic MCM DNA replication proteins
Source: BMC Evol Biol. 2009 Mar 17;9:60. doi: 10.1186/1471-2148-9-60 (PMC2667178; doi:10.1186/1471-2148-9-60)

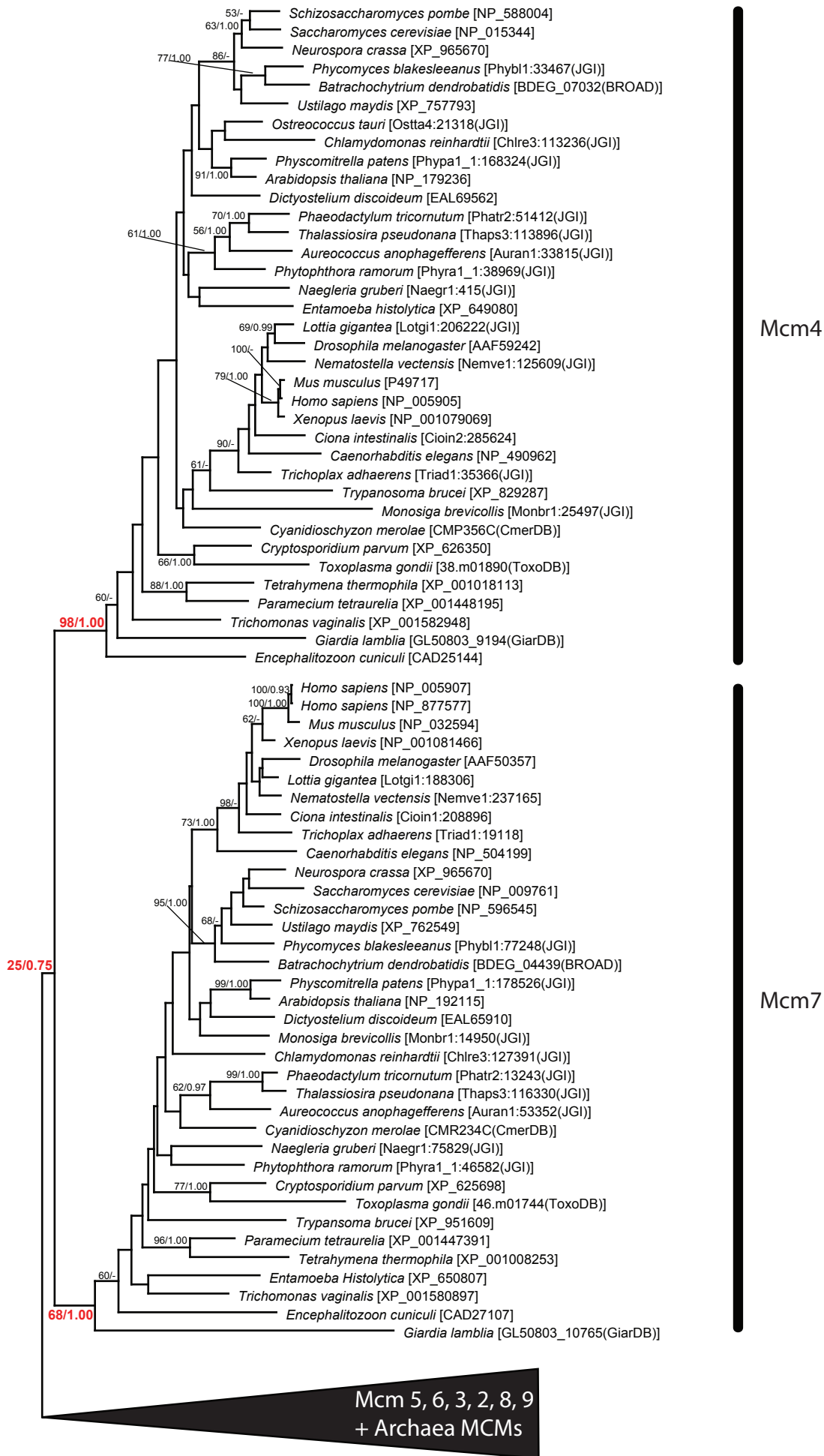

Supplement: Additional file 1 — Phylogenetic analysis of eukaryotic Mcm2-9 (part 1: Mcm4, Mcm7). The tree was generated by fast ML analysis using PHYML and rooted with archaeal MCMs. The numbers on each node are the bootstrap values and posterior probabilities from Bayesian analysis (values below 50% and 0.90 are not shown). Supporting values for each MCM paralogue and for the relationships between the eight MCM paralogues are highlighted in red. [file 1471-2148-9-60-S1.pdf]

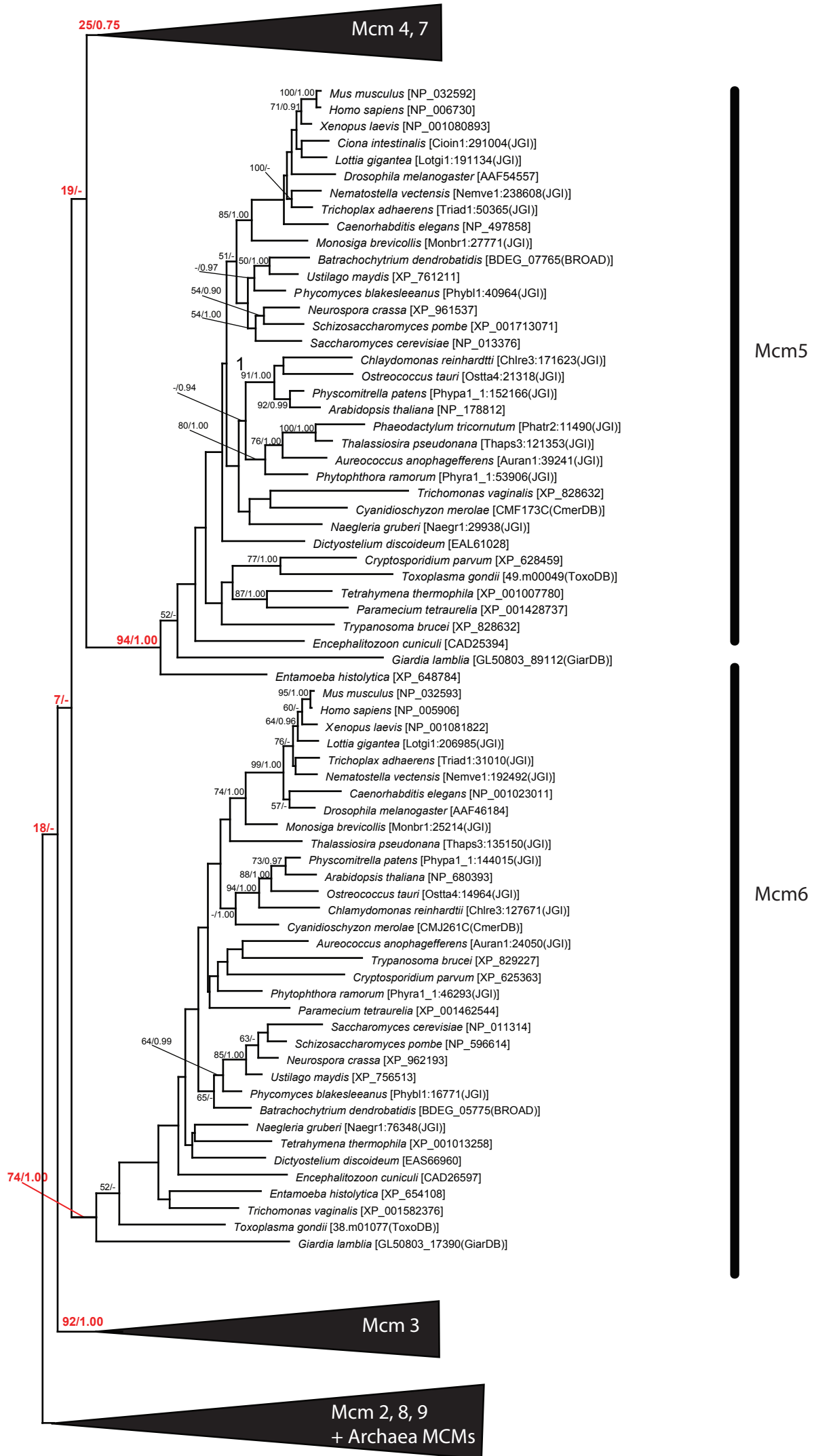

Supplement: Additional file 2 — Phylogenetic analysis of eukaryotic Mcm2-9 (part 2: Mcm5, Mcm6). The tree was generated by fast ML analysis using PHYML and rooted with archaeal MCMs. The numbers on each node are the bootstrap values and posterior probabilities from Bayesian analysis (values below 50% and 0.90 are not shown). Supporting values for each MCM paralogue and for the relationships between the eight MCM paralogues are highlighted in red. [file 1471-2148-9-60-S2.pdf]

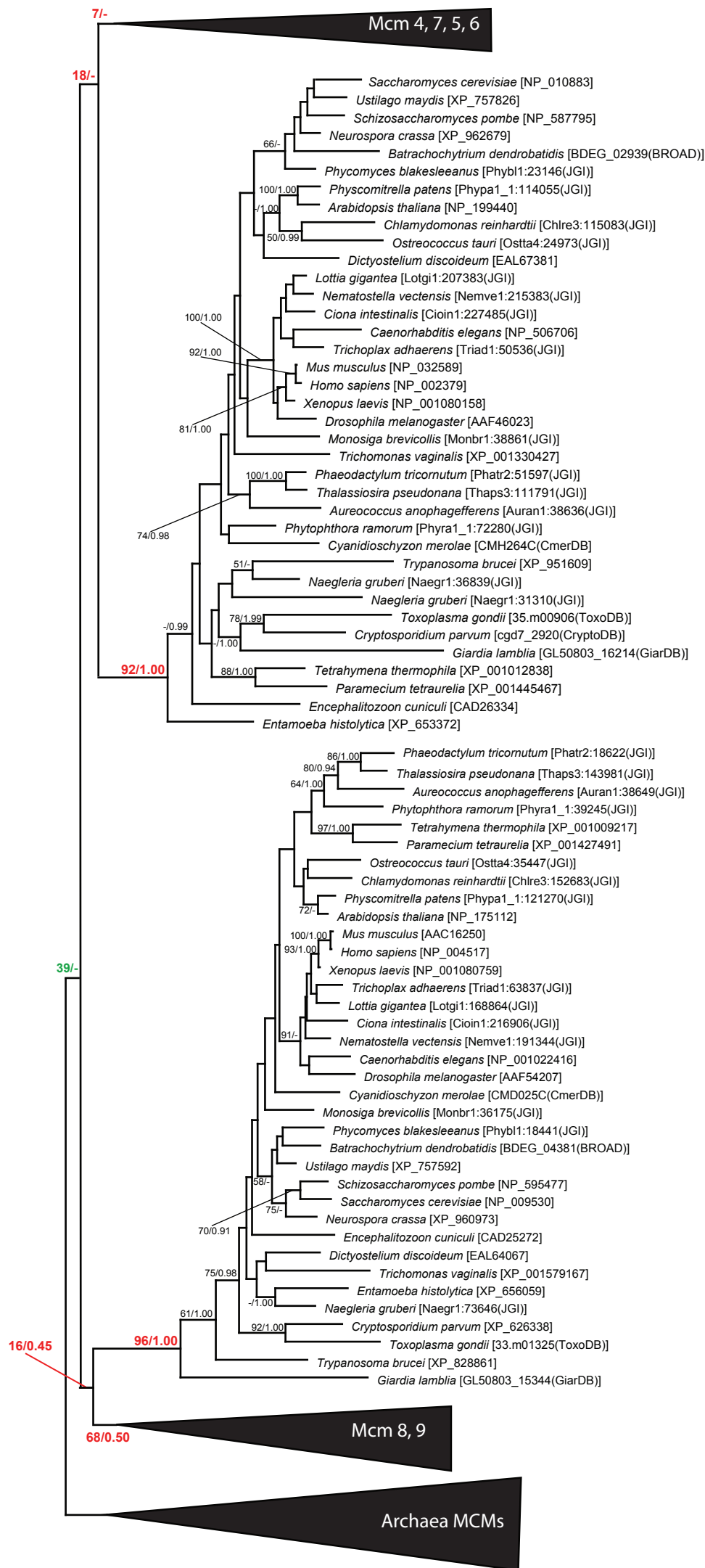

Supplement: Additional file 3 — Phylogenetic analysis of eukaryotic Mcm2-9 (part 3: Mcm3, Mcm2). The tree was generated by fast ML analysis using PHYML and rooted with archaeal MCMs. The numbers on each node are the bootstrap values and posterior probabilities from Bayesian analysis (values below 50% and 0.90 are not shown). Supporting values for each MCM paralogue and for the relationships between the eight MCM paralogues are highlighted in red. Highlighted in green is the supporting value for the origin of eukaryotic MCMs. [file 1471-2148-9-60-S3.pdf]

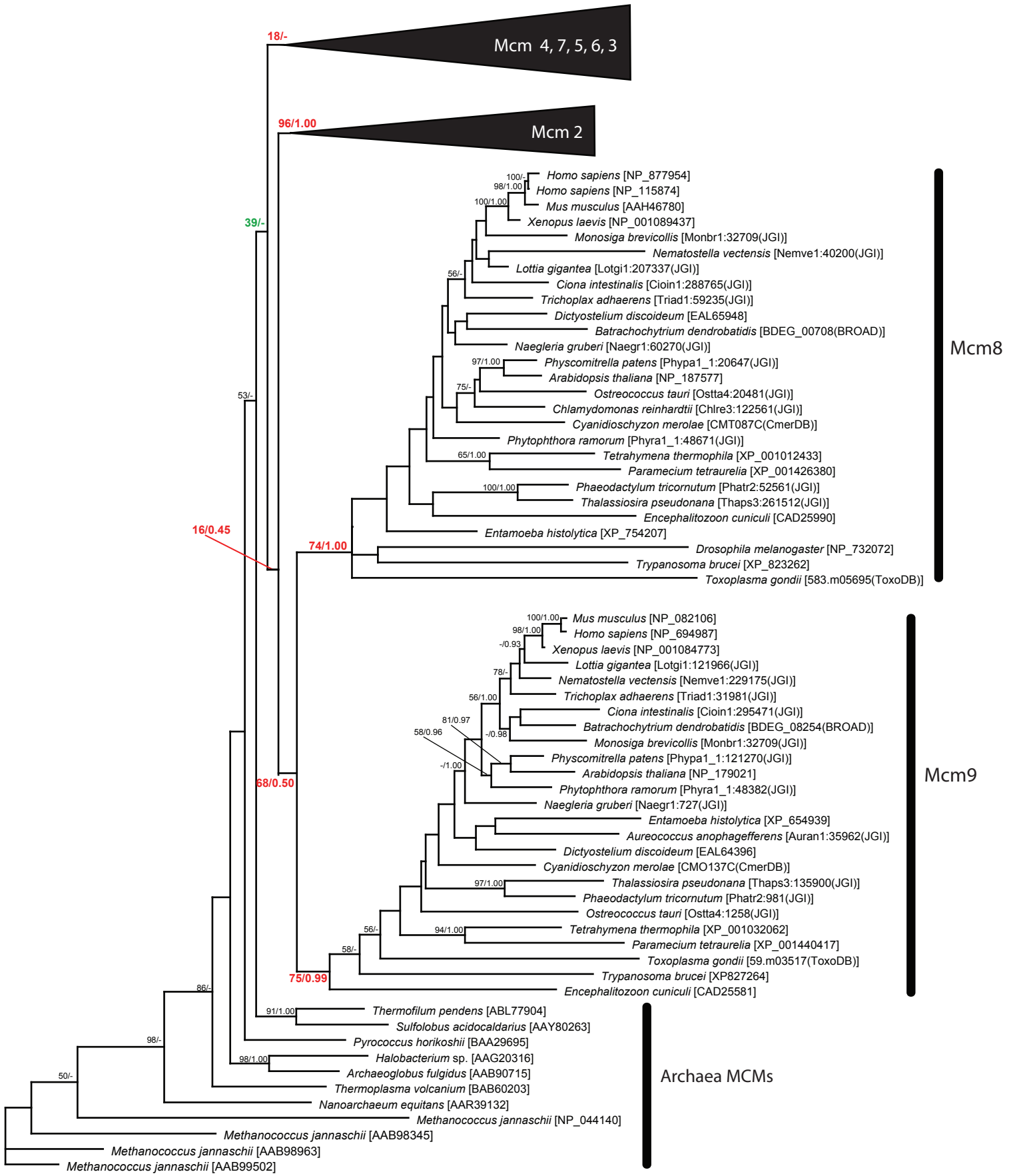

Supplement: Additional file 4 — Phylogenetic analysis of eukaryotic Mcm2-9 (part 4: Mcm8, Mcm9, archaeal MCMs). The tree was generated by fast ML analysis using PHYML and rooted with archaeal MCMs. The numbers on each node are the bootstrap values and posterior probabilities from Bayesian analysis (values below 50% and 0.90 are not shown). Supporting values for each MCM paralogue and for the relationships between the eight MCM paralogues are highlighted in red. Highlighted in green is the supporting value for the origin of eukaryotic MCMs. [file 1471-2148-9-60-S4.pdf]
