# Supplementary material for: Ancient diversification of eukaryotic MCM DNA replication proteins
Source: BMC Evol Biol. 2009 Mar 17;9:60. doi: 10.1186/1471-2148-9-60 (PMC2667178; doi:10.1186/1471-2148-9-60)
Supplement: Additional file 6 — Eukaryotic genomes and predicted proteomes analysed in this study. [file 1471-2148-9-60-S6.doc]

## Additional file 6 - Eukaryotic genomes and predicted proteomes analysed in this study.

| **Species** | **Genome Database (URL)** |
| --- | --- |
| *Dictyostelium discoideum* | dictyBase http://dictybase.org/ |
| *Entamoeba histolytica* | Tiger Database http://www.tigr.org/tdb/e2k1/eha1/new.shtml |
| *Aureococcus anophagefferens* | DOE Joint Genome Institute http://genome.jgi-psf.org/Auran1/Auran1.home.html |
| *Paramecium tetraurelia* | ParameciumDB  http://paramecium.cgm.cnrs-gif.fr/db/index |
| *Tetrahymena thermophila* | Tiger Database http://www.tigr.org/tdb/e2k1/ttg/ |
| *Cryptosporidium* *parvum* | CryptoDB http://cryptodb.org/cryptodb/ |
| *Toxoplasma gondii* | ToxoDB http://www.toxodb.org/toxo/home.jsp |
| *Plasmodium berghei* | PlasmoDB http://www.plasmodb.org/plasmo/home.jsp |
| *Phytophthora ramorum* | DOE Joint Genome Institute http://genome.jgi-psf.org/Phyra1_1/  Phyra1_1.home.html |
| *Phaeodactylum tricornutum* | DOE Joint Genome Institute http://genome.jgi-psf.org/Phatr2/Phatr2.home.html |
| *Thalassiosira pseudonana* | DOE Joint Genome Institute http://genome.jgi-psf.org/Thaps3/  Thaps3.home.html |
| *Naegleria gruberi* | DOE Joint Genome Institute  http://genome.jgi-psf.org/Naegr1/  Naegr1.home.html |
| *Giardia lamblia* | GiardiaDB http://gmod.mbl.edu/perl/site/giardia14?page=intro |
| *Trichomonas vaginalis* | Tiger Database http://www.tigr.org/tdb/e2k1/tvg/ |
| *Trypanosoma brucei* | GeneDB hostby Sanger Institute http://www.genedb.org/genedb/tryp/index.jsp |
| *Arabidopsis thaliana* | The Arabidopsis Information Resource http://www.arabidopsis.org/index.jsp |
| *Physcomitrella patens* | DOE Joint Genome Institute http://genome.jgi-psf.org/Phypa1_1/  Phypa1_1.home.html |
| *Chlamydomonas reinhardtii* | DOE Joint Genome Institute  http://genome.jgi-psf.org/Chlre3/Chlre3.home.html |
| *Ostreococcus tauri* | DOE Joint Genome Institute  http://genome.jgi-psf.org/Ostta4/Ostta4.home.html |
| *Cyanidioschyzon merolae* | Cyanidioschyzon merolae Genome Project http://merolae.biol.s.u-tokyo.ac.jp/ |
| *Caenorhabditis elegans* | Ensembl |
| *Ciona intestinalis* | DOE Joint Genome Institute  http://genome.jgi-psf.org/Cioin2/Cioin2.home.html |
| *Drosophila melanogaster* | FlyBase http://flybase.bio.indiana.edu/ |
| *Mus musculus* | NCBI |
| *Nematostella vectensis* | DOE Joint Genome Institute http://genome.jgi-psf.org/Nemve1/  Nemve1.home.html |
| *Homo sapiens* | NCBI |
| *Lottia gigantea* | DOE Joint Genome Institute  http://genome.jgi-psf.org/Lotgi1/Lotgi1.home.html |
| *Trichoplax adhaerens* | DOE Joint Genome Institute  http://genome.jgi-psf.org/Triad1/Triad1.home.html |
| *Xenopus laevis* | Xenbase http://www.xenbase.org/ |
| *Batrachochytrium dendrobatidis* | Broad Institute http://www.broad.mit.edu/annotation/genome/  batrachochytrium_dendrobatidis |
| *Encephalitozoon cuniculi* | EMBL |
| *Neurospora crassa* | Broad Institute http://www.broad.mit.edu/annotation/genome/  neurospora |
| *Saccharomyces cerevisiae* | GeneDB hostby Sanger Institute http://www.genedb.org/genedb/cerevisiae/ |
| *Schizosaccharomyces pombe* | GeneDB hostby Sanger Institute http://www.genedb.org/genedb/pombe/ |
| *Phycomyces blakesleeanus* | DOE Joint Genome Institute http://genome.jgi-psf.org/Phybl1/  Phybl1.home.html |
| *Ustilago maydis* | Broad Institute http://www.broad.mit.edu/annotation/genome/ ustilago_maydis/Home.html |
| *Monosiga brevicollis* | DOE Joint Genome Institute http://genome.jgi-psf.org/Monbr1/  Monbr1.home.html |

## 
